# Policy analysis and expert consultation on cross-border telehealth services

**DOI:** 10.2471/BLT.25.294599

**Published:** 2026-04-16

**Authors:** Caroline Perrin Franck, Salim Azzabi, Joscelyn Magdeleine, Pierre Sauvé, Derrick Muneene, Alain Labrique

**Affiliations:** a University of Geneva, Campus Biotech, Chemin des Mines 9, 1202 Geneva, Switzerland.; b Department of Data and Analytics, Digital Health and Delivery for Impact, World Health Organization, Geneva, Switzerland.; c Trade in Services and Investment Division, World Trade Organization, Geneva, Switzerland.; d World Trade Institute, University of Bern, Bern, Switzerland.

## Abstract

Evidence suggests telehealth can be cost-effective and improve outcomes, including in cross-jurisdictional settings. We examined how cross-border telehealth could support health system transformation and consulted experts to identify priority policy considerations for integrating services across jurisdictions. We conducted a multimethod policy analysis comprising a narrative review informed by targeted searches and policy mapping, pre-Delphi working-group discussions, and a three-round modified Delphi consensus process with 18 experts in health policy, health-care regulation, telehealth implementation and international trade. We then made our draft findings available for World Health Organization-hosted public consultation, incorporating feedback and updating our narrative review. Our finalized challenge–policy matrix included 20 key challenges that we categorized within seven domains: knowledge and awareness; policy and governance; technology and operational issues; data security and privacy; quality and ethical standards; financial and economic factors; and adaptability and resilience. In our detailed challenge–policy matrix we identify national and international required policy actions for all 20 key challenges, which we summarize as: reinforce regulatory frameworks and international commitments; strengthen governance and coordination; build digital health capacity; ensure data privacy and security; and promote sustainable financing models. From a public health perspective, cross-border telehealth is essential for the strengthening of global health systems. Tackling the challenges of regulatory heterogeneity, exclusion from international trade agreements, digital literacy, language barriers and financial constraints requires that regulatory, technological and operational constraints be addressed through international policy cooperation. Priorities include interoperable regulatory frameworks, reduction or removal of unnecessary barriers, infrastructure investment, data protection, sustainable financing and emergency preparedness.

## Résumé

Les données disponibles indiquent que la télésanté peut être rentable et améliorer les issues médicales, y compris dans des contextes transfrontaliers. Nous avons examiné la façon dont la télésanté transfrontalière pourrait soutenir la transformation du système de santé et avons consulté des experts afin d’identifier les considérations politiques prioritaires pour l’intégration des services entre les juridictions. Nous avons mené une analyse des politiques axée sur plusieurs méthodologies. Celle-ci comprenait un examen narratif s’appuyant sur des recherches ciblées et une identification des politiques, des discussions au sein d’un groupe de travail pré-Delphi, ainsi qu’un processus de consensus axé sur la méthode Delphi modifiée en trois phases réunissant 18 experts en politique de santé, en réglementation des soins de santé, en mise en œuvre de la télésanté et en commerce international. Nous avons ensuite soumis notre projet de conclusions à une consultation publique organisée par l’Organisation mondiale de la Santé, en intégrant les commentaires reçus et en mettant à jour notre examen narratif. Notre matrice finale faisant la part entre les défis et les politiques comprenait 20 défis clés que nous avons classés en sept domaines: connaissances et sensibilisation; politiques et gouvernance; technologie et questions opérationnelles; sécurité et confidentialité des données; normes de qualité et éthiques; facteurs financiers et économiques; ainsi qu’adaptabilité et résilience. Cette matrice détaillée identifie les mesures politiques nécessaires, tant au niveau national qu’international, pour relever l’ensemble des 20 défis clés, que nous résumons comme suit: renforcement des cadres réglementaires et des engagements internationaux; consolidation de la gouvernance et de la coordination; développement des capacités en matière de santé numérique; garantie de la confidentialité et de la sécurité des données; et promotion de modèles de financement durables. Du point de vue de la santé publique, la télésanté transfrontalière est essentielle au renforcement des systèmes de santé mondiaux. Pour relever les défis liés à l’harmonisation réglementaire, à l’exclusion des accords commerciaux internationaux, à la culture numérique, aux barrières linguistiques et aux contraintes financières, il importe de s’attaquer aux contraintes réglementaires, technologiques et opérationnelles au travers d’une coopération politique internationale. Les priorités comprennent l’interopérabilité des cadres réglementaires, la réduction ou la suppression des obstacles inutiles, l’investissement dans les infrastructures, la protection des données, le financement durable et la préparation aux situations d’urgence.

## Resumen

La evidencia sugiere que la telesalud puede ser rentable y mejorar los resultados, incluso en contextos transjurisdiccionales. Se examinó cómo la telesalud transfronteriza podría apoyar la transformación del sistema sanitario y se consultó a expertos para identificar consideraciones prioritarias de política para la integración de servicios entre distintas jurisdicciones. Se llevó a cabo un análisis de políticas con múltiples métodos que incluyó una revisión narrativa informada por búsquedas específicas y mapeo de políticas, debates de grupos de trabajo previos al método Delphi y un proceso de consenso Delphi modificado en tres rondas con 18 expertos en políticas sanitarias, regulación de la asistencia sanitaria, implementación de telesalud y comercio internacional. Posteriormente, los resultados preliminares se pusieron a disposición para una consulta pública organizada por la Organización Mundial de la Salud, incorporando las observaciones recibidas y actualizando la revisión narrativa. La matriz final de retos y políticas incluyó 20 desafíos clave, que se clasificaron en siete ámbitos: conocimiento y sensibilización; políticas y gobernanza; cuestiones tecnológicas y operativas; seguridad y privacidad de los datos; calidad y normas éticas; factores financieros y económicos; y adaptabilidad y resiliencia. En la matriz detallada de retos y políticas se identifican las medidas de política necesarias a nivel nacional e internacional para los 20 desafíos clave, que se resumen en: reforzar los marcos regulatorios y los compromisos internacionales; fortalecer la gobernanza y la coordinación; desarrollar la capacidad en salud digital; asegurar la privacidad y la seguridad de los datos; y promover modelos de financiación sostenibles. Desde una perspectiva de salud pública, la telesalud transfronteriza es esencial para el fortalecimiento de los sistemas sanitarios a nivel mundial. Abordar los desafíos derivados de la heterogeneidad regulatoria, la exclusión de los acuerdos comerciales internacionales, la alfabetización digital, las barreras lingüísticas y las limitaciones financieras requiere que las restricciones regulatorias, tecnológicas y operativas se afronten mediante la cooperación internacional en materia de políticas. Las prioridades incluyen marcos regulatorios interoperables, la reducción o eliminación de barreras innecesarias, la inversión en infraestructuras, la protección de datos, la financiación sostenible y la preparación ante emergencias.

## ملخص

توضح الأدلة أن الطب عن بُعد قد يكون فعالاً من حيث التكلفة ويُحسّن النتائج، بما في ذلك في البيئات عبر الحدود. قمنا بدراسة كيف يمكن للطب عن بُعد عبر الحدود أن يدعم التحوّل في النظام الصحي، واستشرنا خبراء لتحديد الاعتبارات السياسية ذات الأولوية لتكامل الخدمات عبر الحدود. وقمنا بإجراء تحليل متعدد الأساليب للسياسات، يتألف من مراجعة سردية قائمة على عمليات بحث مُوجّهة ورسم خرائط للسياسات، ومناقشات مجموعات العمل التمهيدية لما قبل Delphi، وعملية توافق آراء مُعدّلة من ثلاث جولات باستخدام Delphi مع 18 خبيرًا في السياسات الصحية، وتنظيم الرعاية الصحية، وتنفيذ الطب عن بُعد، والتجارة الدولية. قمنا بعد ذلك بتوفير نتائجنا الأولية للاستشارة العامة التي استضافتها منظمة الصحة العالمية، مع دمج الملاحظات وتحديث مراجعتنا السردية. اشتملت مصفوفة التحديات والسياسات النهائية على 20 تحديًا رئيسيًا صنّفناها ضمن سبعة مجالات: المعرفة والوعي؛ والسياسات والحوكمة؛ والتكنولوجيا والقضايا التشغيلية؛ وأمن البيانات والخصوصية؛ ومعايير الجودة والأخلاقيات؛ والعوامل المالية والاقتصادية؛ والقدرة على التكيف والمرونة. في مصفوفة التحديات والسياسات التفصيلية لدينا، نحدد الإجراءات السياسية الوطنية والدولية المطلوبة لمواجهة جميع التحديات الرئيسية الـ 20، والتي نلخصها في: تعزيز أطر العمل التنظيمية والالتزامات الدولية؛ ودعم الحوكمة والتنسيق؛ وبناء القدرات الصحية الرقمية؛ وضمان خصوصية البيانات وأمنها؛ وتعزيز نماذج التمويل المستدام. ومن منظور الصحة العامة، فإن الطب عن بُعد عبر الحدود يُعدّ أساسيًا لتعزيز النظم الصحية العالمية. إن التعامل مع تحديات التباين التنظيمي، والاستبعاد من اتفاقيات التجارة الدولية، ومحو الأمية الرقمية، والحواجز اللغوية، والقيود المالية، يتطلب معالجة القيود التنظيمية والتكنولوجية والتشغيلية من خلال التعاون السياسي الدولي. وتشمل الأولويات أطر عمل تنظيمية قابلة للتشغيل البيني، والحد من الحواجز غير الضرورية أو إزالتها، والاستثمار في البنية التحتية، وحماية البيانات، والتمويل المستدام، والاستعداد للطوارئ.

## 摘要

有证据表明,远程卫生保健服务的成本效益较高,并能显著改善结局(包括在跨司法管辖区的情况下)。我们研究了跨境远程卫生保健服务是如何能够支持卫生系统转型的,并咨询了多位专家以确定跨司法管辖区整合服务时需考虑的优先政策事项。我们进行了多方法政策分析,包括基于定向搜索和政策映射的叙述性综述、特尔菲前期工作组讨论以及由来自卫生政策、卫生保健监管、远程卫生保健服务履行和国际贸易领域的 18 位专家共同制定的三轮改良特尔菲共识法。然后,我们将初步研究结果提交给世卫组织并由其开展公众咨询活动,从而采纳反馈意见并更新我们的叙述性综述。我们最后敲定的挑战-政策矩阵包含了归入以下七个领域的 20 项关键挑战:知识与意识;政策与治理;技术与运营问题;数据安全与隐私;质量与道德标准;财务因素与经济因素;以及适应性与韧性。在我们的详细挑战-政策矩阵中,我们针对所有 20 项关键挑战确定了符合各国要求及国际要求的政策行动,我们将此类行动汇总如下:强化监管框架和切实履行国际承诺;加强治理和协调;构建数字卫生能力;保障数据隐私和安全;以及推广可持续筹资模式。从公共卫生的角度看,跨境远程卫生保健服务对强化全球卫生系统而言至关重要。如要应对监管异质性、被排除在国际贸易协定之外、数字素养不足、语言障碍和财务限制等挑战,必须借助国际政策合作解除监管、技术和运营方面的限制。优先事项包括构建可互操作监管框架、减少或清除不必要的障碍、进行基础设施投资、提供数据保护、推广可持续筹资以及做好应急准备。

## Резюме

По имеющимся данным, телемедицина может быть экономически выгодным вмешательством, улучшающим результаты лечения, в том числе в ситуациях со смешанной юрисдикцией. Авторы изучили, каким образом трансграничная телемедицина может способствовать трансформации систем здравоохранения, и проконсультировались с экспертами для определения приоритетных вопросов политики при интеграции услуг в различных юрисдикциях. Был выполнен многоэтапный анализ политики с использованием нескольких методов, который включал: обзор литературы на основе целевых поисковых запросов и картирования политики, предварительные обсуждения в рабочих группах до использования метода Delphi, а также трехраундовый модифицированный процесс достижения консенсуса Delphi с участием 18 экспертов в области политики здравоохранения, регулирования здравоохранения, внедрения телемедицины и международной торговли. Затем авторы представили предварительные результаты для общественного обсуждения под эгидой Всемирной организации здравоохранения, учли полученные замечания и обновили обзор литературы. В итоговую матрицу «проблема-политика» вошли 20 ключевых проблем, которые были распределены по семи категориям: знания и осведомленность; политика и управление; технологические и операционные вопросы; безопасность и конфиденциальность данных; стандарты качества и этики; финансово-экономические факторы; адаптивность и устойчивость. В подробной матрице «проблема-политика» авторы определили необходимые на национальном и международном уровнях политические меры для решения всех 20 ключевых проблем, которые можно обобщить следующим образом: укрепление нормативно-правовой базы и международных обязательств; усиление управления и координации; развитие цифрового потенциала сферы здравоохранения; обеспечение конфиденциальности и безопасности данных; продвижение устойчивых моделей финансирования. С точки зрения общественного здравоохранения трансграничная телемедицина имеет важнейшее значение для укрепления глобальных систем здравоохранения. Решение проблем, связанных с разнородностью законодательной базы, исключением из международных торговых соглашений, цифровой грамотностью, языковыми барьерами и финансовыми ограничениями, требует устранения нормативных, технологических и операционных барьеров посредством международного сотрудничества в сфере политики. К приоритетным направлениям относятся: создание совместимых нормативно-правовых механизмов, сокращение или устранение ненужных барьеров, инвестиции в инфраструктуру, защита данных, устойчивое финансирование и готовность к чрезвычайным ситуациям.

## Introduction

Telehealth, the delivery of health services at distance via information and communication technologies, has expanded rapidly in recent years, accelerated by technological advances, economic growth and the occurrence of the coronavirus disease 2019 (COVID-19) pandemic.^1^^–^^6^ Utilization to support continuity of care increased exponentially during the pandemic (by an estimated 38-fold),^7^ with particularly strong uptake in specialities suited to remote delivery such as radiology, pathology and peer-to-peer specialist consultations.^8^^–^^10^ Evidence suggests telehealth can be cost-effective and improve outcomes, including in cross-jurisdictional settings.^5^^,^^6^^,^^8^^–^^16^

A growing subdomain is cross-border telehealth, where patients, providers and/or supporting platforms operate in different jurisdictions. In practice, cross-border telehealth is most common in services deliverable at distance with limited physical interaction. Illustrative examples include: peer-to-peer tele-consultative critical care services linking specialists to remote facilities in Afghanistan, Kenya, Pakistan and United Republic of Tanzania; and telehealth deployments supporting international mass gatherings such as the Hajj, during which Saudi Arabia’s Seha Virtual Hospital provided nonstop, round-the-clock multilingual telehealth services for millions of pilgrims.^17^^–^^19^ Language barriers are typically managed through bilingual clinicians, interpreter-supported workflows and digital translation tools.^17^^,^^20^

Global health spending reached 9.8 trillion United States dollars (US$) in 2021,^21^ so even modest growth in digitally delivered services across borders can have material implications for access, workforce distribution and resilience, particularly in underserved settings and during emergencies.^1^^,^^22^^,^^23^ Although cross-border telehealth (categorized within international trade in services) represents a small proportion of overall health service delivery, its significance is increasing. Trade in medical services is sizeable (global exports of health services totalled US$ 94.4 billion in 2022),^24^ and digitally delivered cross-border services, including telehealth, expanded during the COVID-19 pandemic even as travel-related health trade declined.^25^ Market estimates suggested rapid growth in cross-border telehealth to around US$ 8 billion in 2022, an increase of approximately sixfold since 2005.^24^

According to World Trade Organization (WTO) data, in 2022 the leading exporters of cross-border telehealth services included the European Union, Australia, Cuba, India, Singapore, United Arab Emirates and United States of America, and the leading importers included the European Union, India, Norway, Qatar, Singapore, United Kingdom of Great Britain and Northern Ireland, and the USA.^24^ These patterns provide an approximation of where cross-border provision and demand are concentrated.^24^^,^^25^

However, realizing the benefits of cross-border telehealth at scale requires regulatory and governance readiness. Insufficient, uncoordinated or unclear frameworks can undermine quality of care, patient safety, data protection and equity.^2^^,^^3^^,^^12^^,^^15^^,^^25^^,^^26^ Cross-border provision is complicated by inconsistent rules on licensing and oversight, liability, prescribing, privacy and cross-border data transfers, reimbursement and payment processes, and uneven infrastructure capacity, sometimes compounded by unnecessary barriers to cross-border supply.^15^^,^^27^^–^^30^ As telehealth expands, these gaps show that the main constraint on cross-border telehealth is not technology but fragmented policy, governance and financing across countries. These challenges must be resolved to unlock the full potential of telehealth in advancing relevant sustainable development goals and universal health coverage (UHC).^22^^,^^31^^,^^32^

In this context, our objectives were to examine how cross-border telehealth could support health system transformation, and to consult experts to identify priority policy considerations for integrating services across jurisdictions. 

## Review and consultation process

### Narrative review and policy mapping

We defined cross-border telehealth as remote clinical service delivery where the patient (or requesting provider) and the consulting provider are in different legal jurisdictions. We focused on digitally delivered cross-border supply, including synchronous consultations, asynchronous store-and-forward services (e.g. teleradiology and/or telepathology) and decision-support models (e.g. tele-intensive care), and considered enabling services (connectivity, platforms and digital identification and/or certification tools) where they affected feasibility, safety or scalability.^16^^,^^25^

We conducted a narrative review informed by targeted searches. We searched the databases PubMed® and Google Scholar in March 2023 for English-language articles published since January 2021 (Box 1). We included publications addressing cross-border telehealth and/or telemedicine and relevant policy, legal or regulatory considerations. We excluded publications with no cross-border dimension (regional or international), conference abstracts without full text and non-health cross-border digital services. After sorting for relevance, we screened all PubMed® results and the first 50 results from Google Scholar.

Box 1 **Search terms used in narrative review of cross-border telehealth and relevant policy**

- PubMed®: (telehealth[tiab] OR telemedicine[tiab] OR telemedicine[mesh]) AND (“cross-border”[tiab] OR crossborder[tiab] OR “cross border”[tiab] OR transnational[tiab] OR international[tiab]) AND (licen*[tiab] OR recognit*[tiab] OR liabilit*[tiab] OR prescrib*[tiab] OR reimbursement[tiab] OR “data protection”[tiab] OR cybersecurity[tiab] OR interoperab*[tiab] OR “trade in services”[tiab] OR GATS[tiab])
- Google Scholar: (telehealth OR telemedicine) (cross-border OR crossborder OR cross border) (policy OR regulation OR legal)

We supplemented these results by searching the websites of WHO, the International Telecommunication Union (ITU), WTO, the World Bank and other national regulators in March 2023 for articles published since January 2021. 

Finally, we mapped relevant regulatory information and trade-related commitments affecting digitally delivered services to identify policy levers pertinent to cross-border telehealth. We used publicly available regulatory and services trade data, including the World Bank–WTO Services Trade Policy Database and WTO commitments data sets, accessible via the World Bank–WTO Integrated Trade Intelligence Portal Services.^33^

### Pre-Delphi working group discussions

We held two semi-structured hybrid group discussions with participants of the WHO telehealth working group (“One-stop shop for telehealth”) in September 2022 (five participants) and October 2022 (four participants), each lasting around 45 minutes. Guided by pre-defined questions, these sessions served as a pre-Delphi scoping step to identify perceived gaps in, barriers to and enablers of cross-border telehealth. Participants contributed in their professional capacity as organizational representatives (two clinicians, five programme managers or implementers, one regulator and one payer). We took notes as opposed to recording the discussions, and did not collect or discuss any patient data. We informed participants of the purpose of the activity, the voluntary nature of participation, and how inputs would be used and reported. We obtained verbal consent from participants at the start of each working group discussion and Delphi round.

### Delphi consensus process and public consultation

To incorporate stakeholder perspectives, we then applied a modified three-round Delphi consensus process guided by the Walt–Gilson policy triangle (context, content, process and actors).^34^

We recruited 18 Delphi panellists through organizational nominations supplemented by purposive identification and snowball referrals, targeting expertise in telehealth implementation, regulation, financing, data protection and cybersecurity, and trade-in-services policy. We aimed for diversity across regions (with 13, three and two panellists from high-, middle- and low-income countries, respectively) and expertise domains in panellist recruitment. Our panellists included nine participants from international organizations (WHO, WTO, ITU and the World Bank), reflecting our focus on normative and policy processes for cross-border telehealth, while the other nine participants represented regulators, telehealth service providers, implementers and academia. 

In Round 1 of the modified Delphi process, panellists provided qualitative feedback on the draft matrix and we revised items accordingly. In Round 2, panellists rated each item for relevance on a 1–5 Likert scale; consensus was defined a priori as an interquartile range of 2 or lower. All items met this criterion (online repository).^35^ In Round 3, we held two hybrid workshops to resolve outstanding issues and finalize our draft matrix. 

After completion of the Delphi process, we published our draft matrix on the WHO website for public consultation (4–25 Apr 2024); of the 95 responses received, 12 included substantive content comments that informed our final revisions. In consultation with our Delphi panellists, we incorporated this feedback, conducted targeted update searches of the literature and relevant organization websites (early 2025), and refined our finalized policy matrix. 

### Ethical considerations 

The pre-Delphi working group discussions and Delphi process were consensus-building activities with experts and organizational representatives acting as partners in decision-making rather than research participants.

## Key challenges identified

We identified 20 key challenges, which we categorized within seven overarching thematic domains that are critical to the implementation of cross-border telehealth (online repository).^35^ These domains and key challenges are summarized in Box 2. The panel emphasized that the policy and governance domain underpins many other challenges. Without clear rules for cross-border practice, including licensing or recognition of qualifications, liability and reimbursement, and the removal of unnecessary barriers, technology and funding alone cannot advance cross-border telehealth. Similarly, data privacy concerns affect public trust (awareness), legal compliance (governance) and the willingness of institutions to participate in cross-country exchanges, cutting across several of the thematic domains listed in Box 2.

Box 2 **The 20 identified key challenges, categorized within seven domains, affecting the implementation of cross-border telehealth**

***Knowledge and awareness***

(i) A limited understanding of the benefits and modalities, combined with (ii) significant digital health literacy gaps, represent key challenges to the adoption of cross-border telehealth.^36^

***Policy and governance***

(iii) Many countries have outdated, inadequate or no legislation, making it challenging to (iv) navigate the regulatory environment and (v) recognize the qualifications and licences of health workers from other jurisdictions in the context of cross-border telehealth.^30^^,^^37^ (vi) Many jurisdictions also maintain unnecessary barriers; the fragmentation of regulations and absence of clear legal frameworks hinder cross-border telehealth.^29^ (vii) Cross-border liability remains unclear, and both (viii) billing and payment mechanisms and (ix) cross-border prescribing are constrained by divergent rules across jurisdictions. (x) Concerns about budget impact and fiscal sustainability may also limit the adoption of cross-border telehealth.

***Technology and operational issues***

(xi) Technological challenges include the limited interoperability of systems and electronic health records across borders. There is also unequal availability of telehealth technology infrastructure. (xii) Ongoing cybersecurity threats, coupled with (xiii) the increasing use of artificial intelligence, have introduced new operational challenges.

***Data security and privacy***

(xiv) Transmitting sensitive health data across national boundaries raises privacy concerns, and cross-border telehealth can be hindered by uncertainty over which jurisdiction’s privacy regulations apply.^38^

***Quality and ethical standards***

Ensuring consistent quality of care in telehealth across countries is challenging when (xv) ethical norms and (xvi) standards of clinical practice differ. (xvii) There may be concerns about diagnostic accuracy, continuity of care and accountability in virtual services. (xviii) Issues of equity may arise if only certain populations can access cross-border care.

***Financial and economic factors***

(xix) Sustainable financing remains a concern. Business models for cross-border telehealth are still in development, and uncertainties remain regarding reimbursement for providers and affordability for patients.^39^ Encouraging foreign investment in the sector, or insurance coverage for telehealth services provided from abroad, could support the development of cross-border telehealth.

***Adaptability and resilience***

(xx) The value of telehealth in maintaining health-care delivery during emergencies was highlighted during the coronavirus disease 2019 pandemic, but not all health systems were prepared to leverage cross-border support.^40^ Fragmented regulations and a lack of infrastructure can prevent telehealth from being scaled up rapidly across borders in response to health emergencies or natural disasters.

## Required policy actions

For each identified challenge, we formulated suggested policy actions at the national and international levels. We list our key cross-cutting policy actions in Box 3; the full set of suggested policy actions is available in the online repository.^35^

Box 3 **Required policy actions (and related key challenges addressed) for the enablement and enhancement of cross-border telehealth**

- Reinforce regulatory frameworks and international commitments (iii–vii). Governments should establish legislation addressing cross-border telehealth practice to clarify the applicable jurisdiction (including standards of care) and how malpractice liability is handled. Governments should also incorporate cross-border telehealth into bilateral and/or regional cooperation arrangements and, where relevant, into trade-in-services commitments to improve transparency and predictability. Professional regulators should develop and adopt procedures to recognize telehealth services and provider licences across jurisdictions, including through mutual recognition agreements.
- Strengthen governance and coordination (iii–vi). Governments should designate national regulatory authorities or focal points for telehealth to streamline oversight. International organizations such as WHO and ITU can facilitate the creation of model regulations and promote the adoption of best practices across countries.
- Build digital health capacity (ii, xi, xviii, xx). Governments should invest in connectivity and/or platform readiness and workforce training, strengthen population digital health literacy and mobilize international support to narrow the digital divide. Development partners and international funding mechanisms may be required to help less-developed health systems bridge the digital divide.
- Ensure data privacy and security (xi, xii, xiv). Interoperable standards to exchange health data and the mutual recognition of data protection regimes are needed. For instance, governments could adopt data protection agreements, enabling the secure transfer of personal health data under defined safeguards. To maintain patient trust and confidentiality in cross-border exchanges, it is important that regulators establish health data stewardship entities or certify telehealth providers for compliance with global privacy standards. International telehealth collaboration should include cybersecurity protocols and information-sharing about cyber threats.
- Promote sustainable financing models (viii, x, xix). Suppliers of health insurance should be encouraged to cover telehealth consultations, including those delivered by overseas providers where appropriate, to ensure financial viability. As part of UHC efforts, governments could consider subsidizing cross-border telehealth for remote or underserved populations. Public–private partnerships can also mobilize resources and innovation; for example, telecoms companies could partner with health-care providers to expand telehealth networks. Finally, governments should create conditions that attract private (foreign) investment.

ITU: International Telecommunication Union; UHC: universal health coverage; WHO: World Health Organization.

The policy considerations listed in Box 3 (and in detail in the online repository)^35^ identify areas where national and international action is needed to enable safe and equitable cross-border telehealth. 

## Discussion

Regulatory readiness for cross-border telehealth remains limited. Many jurisdictions lack explicit provisions for accepting services from abroad; for example, Austria has no specific telehealth legislation and generally restricts foreign doctors to exceptional circumstances and collaboration with a locally licensed physician.^41^ Such practices, which are common in several jurisdictions, reflect concerns about quality control and accountability when care is delivered from abroad. Although Brazil’s telehealth framework is comprehensive for domestic services, it mandates that telehealth providers be incorporated entities in Brazil and register with national medical boards.^42^^–^^44^ Similarly, Bahrain has embraced telehealth, but requires remote providers to obtain a local licence or authorization, ensuring necessary regulatory oversight.^45^ These requirements effectively limit direct cross-border provision. Regulatory heterogeneity means that a doctor or telehealth company approved in one jurisdiction may encounter legal obstacles when supplying services in another. Our recommendations for mutual recognition arrangements and converging standards directly address this challenge, and align with broader calls in global health for regulatory convergence to support the scaling-up of telehealth.

Another critical insight is the interplay between cross-border telehealth and international trade policy. Cross-border telehealth services represent a form of trade in services (specifically via Mode 1 of the WTO General Agreement on Trade in Services) and relevant preferential trade agreements, in which the health service digitally crosses the border.^46^ Such agreements exclude services supplied in the exercise of governmental authority, that is, not supplied on a commercial basis and not in competition. Health-related services have attracted limited attention in trade-in-services negotiations, and telehealth is rarely addressed explicitly in commitments. This policy gap suggests an opportunity: international trade agreements (at the multilateral, regional or bilateral levels) could incorporate commitments to facilitate cross-border telehealth. For example, explicitly discriminatory, unnecessary or needlessly burdensome restrictions on telehealth services could be progressively reduced or removed, and discussions on the mutual accreditation of providers could be encouraged. A recent joint report by global institutions emphasizes the need for cooperation on trade in health services to strengthen pandemic preparedness.^25^ Cross-border telehealth could benefit from deepened international cooperation, and represents an important area for policy development to ensure that trade agreements promote telehealth while respecting the rights of governments to regulate health care.

The policy questions addressed by our Delphi process align with the major structural trend of digitization of services trade and growing reliance on remote expertise, even though measurement of telehealth-specific flows is still limited.^25^ Telehealth can improve access, quality and efficiency of care.^2^^,^^3^^,^^5^^,^^47^ Cross-border telehealth can support low-resource settings by linking providers to specialist care and training; telehealth partnerships have delivered continuing medical education and specialist consultations for rural clinicians.^16^ However, these benefits depend on digital literacy and community awareness. Telehealth will not reduce inequities if patients and health workers cannot access or use it, reinforcing calls for multistakeholder action to bridge the digital divide.^27^

Frontline and patient evidence aligns with the priorities of our Delphi panellists: studies of people facing language barriers show lower video uptake and greater reliance on audio-only visits, emphasizing the need for integrated interpretation and user-centred design. In the WHO Region of the Americas, a study commissioned by the Inter-American Development Bank found limited use of international telemedicine, identifying remuneration, governance and regulatory fragmentation as key constraints. Together, these findings support multilingual service design, transparent pricing and/or consent, and reimbursement clarity (online repository).^20^^,^^35^^,^^48^

Financing and compensation constraints are also evident within countries: reimbursement rules, coverage and payment parity vary across jurisdictions and payers, and licensing requirements can differ within countries with federal systems of governance. These domestic complexities are amplified in cross-border telehealth, reinforcing the need for clear reimbursement policies and interoperable billing standards as part of cross-border regulatory design.^29^^,^^49^

From a public health perspective, addressing these challenges is essential to integrate telehealth into UHC and health system strengthening.^1^ Real-world examples show the feasibility of cross-border telehealth in high-demand settings, and WHO is piloting global digital health certification tools to support cross-border coordination and verification.^19^ These illustrate how infrastructure, regulatory preparedness and international collaboration can enable cross-border telehealth during emergencies and mass events.

Universities and academic networks can play a catalytic role by embedding digital health competencies in pre-service curricula, supporting continuing professional development, and partnering with health systems to evaluate cross-border telehealth models and build trust through evidence generation.^16^

We acknowledge the limitations of our analysis. The telehealth landscape is evolving rapidly; policies and technologies are continuing to change in the post-pandemic era. Our analysis provides a snapshot based on the latest available data and expert input, but the policies of some countries may have changed since the end of our study. Furthermore, although our expert panel was diverse and international, it may not have captured all viewpoints. For example, patient advocacy perspectives may differ in terms of priorities. Although we aimed for regional and disciplinary diversity, the pool of experts with cross-border telehealth policy experience is relatively limited, which may have influenced panel composition and the range of perspectives captured. To mitigate this, we complemented our Delphi process with an open public consultation.

The feasibility of implementing certain recommendations, such as international regulatory convergence, remains to be tested, as political will is required and resource constraints could pose challenges. Despite these limitations, our study benefited from our systematic approach. By combining literature, real-world insights and consensus-building, we have advanced a coherent set of policy considerations that are grounded in literature and expert judgement.

Cross-border telehealth can improve access and strengthen health systems, but requires coordinated action on regulation, digital capacity, data protection and sustainable financing. International collaboration, through knowledge sharing, standards and supportive trade agreements, is critical to embed cross-border telehealth in health-care strategies, and accelerate progress towards UHC and health security.
